# A Rare Histologic Variant of Thymoma: Diagnostic Challenges of Micronodular Thymoma With Lymphoid Stroma

**DOI:** 10.7759/cureus.112033

**Published:** 2026-07-04

**Authors:** Chaimae Bekhakh, Hamraoui Salima, Anass Haloui, Nassira Karich, Amal Bennani

**Affiliations:** 1 Department of Pathology, Mohammed VI University Hospital, Faculty of Medicine and Pharmacy of Oujda, Mohammed First University of Oujda, Oujda, MAR; 2 Department of Thoracic Surgery, Mohammed VI University Hospital, Faculty of Medicine and Pharmacy of Oujda, Mohammed First University, Oujda, MAR

**Keywords:** anterior mediastinal mass, immunohistochemistry, lymphoid stroma, micronodular thymoma, thymic neoplasm

## Abstract

Micronodular thymoma with lymphoid stroma (MNT) is a rare subtype of thymoma characterized by multiple epithelial micronodules embedded within abundant lymphoid stroma containing prominent germinal centers. It generally exhibits an indolent clinical course and a favorable prognosis after surgical excision. We report the case of a 64-year-old woman who presented with an anterior mediastinal mass. Histopathological examination demonstrated micronodular proliferation of spindle-to-oval epithelial cells associated with dense lymphoid stroma and reactive germinal centers. Immunohistochemical study showed positivity of tumor cells for cytokeratin, while CD20-positive B lymphocytes were predominantly localized within follicles, and TdT-positive immature T lymphocytes surrounded the epithelial nests. The tumor cells were negative for CD5 and CD20. Based on the morphological and immunophenotypic findings, a diagnosis of micronodular thymoma with lymphoid stroma was established. This report highlights the characteristic histopathological features of this uncommon entity and emphasizes the importance of differentiating it from other thymic epithelial tumors and micronodular thymic carcinoma. Complete surgical excision remains the treatment of choice and is usually associated with an excellent prognosis.

## Introduction

Thymomas represent the most common primary tumors of the anterior mediastinum in adults, encompassing a heterogeneous group of epithelial neoplasms classified by the World Health Organization (WHO) into subtypes A, AB, B1, B2, B3, and several rare variants, based on the morphology of neoplastic epithelial cells and the relative proportion of intratumoral lymphocytes [1].

Among these rare variants, micronodular thymoma with lymphoid stroma (MNT) represents an exceptionally distinctive subtype, accounting for 1-5% of all thymomas according to the WHO classification [1], first formally characterized by Suster and Moran in 1999 [2] and subsequently incorporated into the WHO classification of thoracic tumors. MNT is defined by its unique biphasic architecture: discrete micronodules of bland epithelial cells set within an abundant lymphoid stroma that frequently harbors reactive follicles with germinal centers, a feature that can lead to misdiagnosis as extranodal lymphoma or other thymic tumors if not carefully evaluated.

To date, approximately 100 cases have been reported in the English-language literature worldwide, predominantly affecting adults in the fifth to seventh decades of life, with a slight male predominance [3]. Given its rarity and deceptively benign histomorphology, MNT may be underdiagnosed or misclassified. We present a case of MNT in a 64-year-old woman with a complex medical background and provide a review of its clinicopathological features, differential diagnosis, and management.

## Case presentation

A 64-year-old woman with no history of myasthenia gravis was referred to the thoracic surgery department following the incidental discovery of an anterior mediastinal mass during an examination to determine the cause of her deep vein thrombosis (DVT). She reported no respiratory symptoms, no chest pain, and no general symptoms such as fever, night sweats, or unintentional weight loss.

A chest CT scan revealed a well-defined, solid-cystic mass located in the anterior mediastinum, measuring approximately 5.8 x 4.5 x 3 cm, with no signs of invasion of adjacent structures, mediastinal lymphadenopathy, or pleural effusion. Staging workup revealed no distant metastatic lesions (Figure 1).

**Figure 1:**
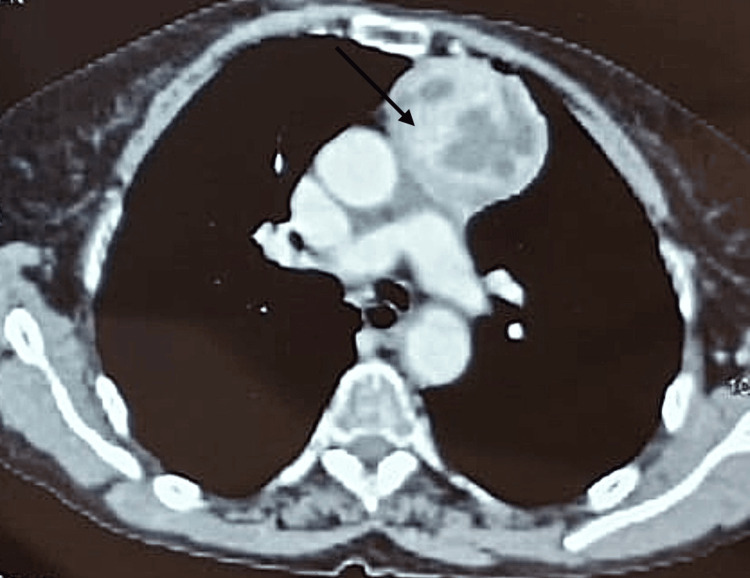
Chest CT scan (axial view) showing a well-defined, solid-cystic mass (arrow) located in the anterior mediastinum.

The patient underwent elective surgical resection via median sternotomy. Intraoperatively, the tumor was found to be well-encapsulated, with no macroscopic signs of capsular rupture or invasion of surrounding structures. A total thymectomy was performed.

Macroscopic examination of the specimen revealed a relatively well-defined solid-cystic thymic mass measuring approximately 6 x 4 x 2.5 cm. The external surface was smooth and surrounded by a fibrous pseudocapsule. The cut surface revealed a predominantly solid component, whitish in color and fleshy in consistency, associated with cystic areas containing clear fluid. No hemorrhage or necrosis was identified in the solid component. The surgical resection margins were macroscopically clear (Figure 2).

**Figure 2:**
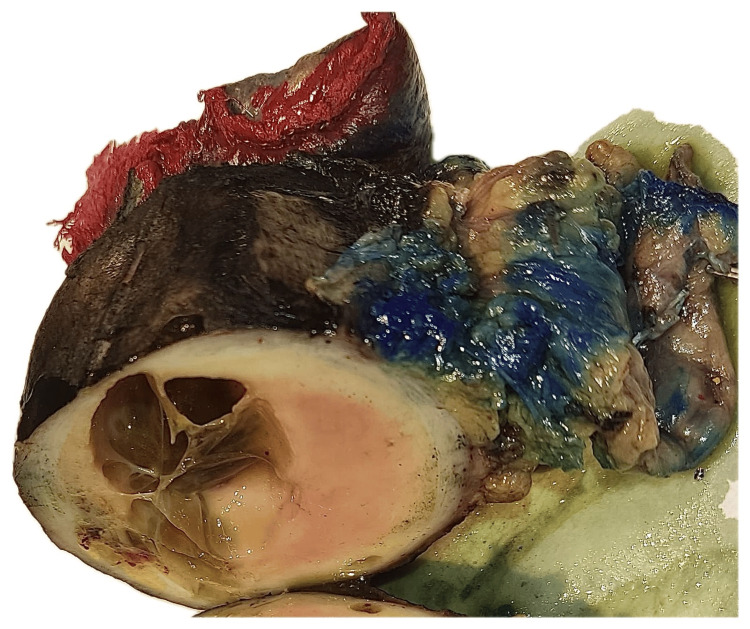
Gross pathology specimen showing a well-encapsulated, solid-cystic mass with a smooth fibrous capsule and cystic spaces.

Microscopic examination of sections revealed a solid thymic lesion with a distinctive biphasic architecture. At low magnification, discrete epithelial micronodules were embedded within an exuberant lymphoid stroma. In certain areas, these nodules exhibited confluence, forming cord-like structures and fused aggregates with a flocculent growth pattern. At higher magnification, the epithelial cells were oval to spindle-shaped, with pale eosinophilic cytoplasm, bland nuclear features, finely dispersed chromatin, and inconspicuous nucleoli. No cytological atypia, mitotic figures, hemorrhage, or necrosis were identified. Scattered lymphocytes were present within the epithelial nodules, while lymphocytes richly populated the intervening stroma. Notably, well-formed lymphoid follicles with reactive germinal centers surrounded by distinct mantle zones were present within the stroma, a hallmark feature of MNT (Figure 3).

**Figure 3:**
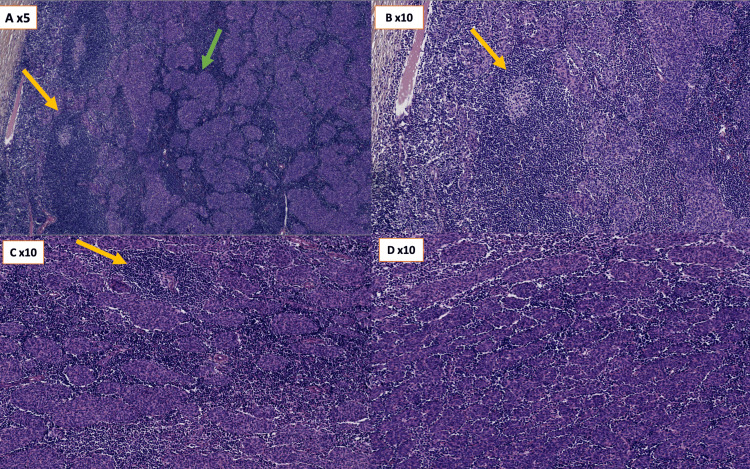
Hematoxylin and eosin staining (A: ×5; B, C, D: ×10) illustrating micronodular proliferation of epithelial cells (green arrow) set within a dense lymphoid stroma containing reactive germinal centers (orange arrows).

An immunohistochemical panel was used to characterize all tumor compartments. The epithelial cells forming the micronodules showed diffuse positive staining for pan-cytokeratin, confirming their thymic epithelial origin. They were negative for the CD5 and CD20 markers. The immature T lymphocytes present in the inter-nodular spaces were positive for the TdT and CD3 markers. CD20 revealed B lymphocytes exclusively in the follicular areas, while mature CD3-positive T lymphocytes were present in the interfollicular stroma (Figure 4).

**Figure 4:**
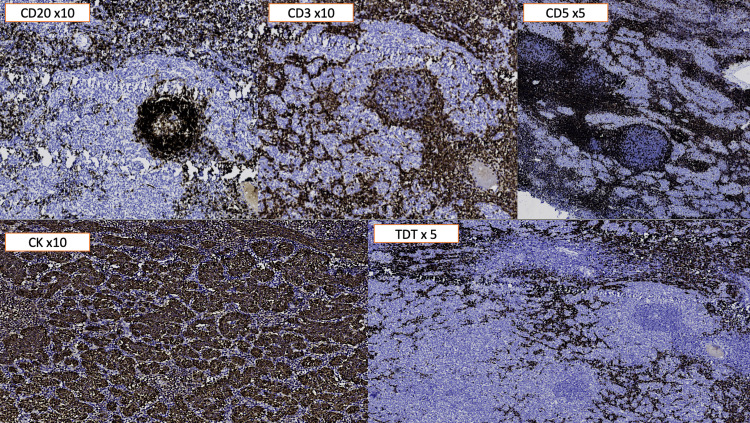
Immunohistochemical profile of micronodular thymoma with lymphoid stroma. CD20 (×10): B lymphocytes predominantly localized within reactive germinal centers. CD3 (×10): T lymphocytes diffusely distributed within the lymphoid stroma surrounding the epithelial micronodules. CD5 (×5): negative expression in tumor epithelial cells. Cytokeratin (CK) (×10): diffuse strong positivity of the epithelial micronodules. TdT (×5): positive immature T lymphocytes surrounding the epithelial nests, negative in tumor cells.

Based on these macroscopic, microscopic, and immunohistochemical findings, a definitive diagnosis of micronodular thymoma with lymphoid stroma (MNT type according to the WHO) was established.

## Discussion

Micronodular thymoma with lymphoid stroma is a rare and histologically distinctive thymic neoplasm first characterized by Suster and Moran in 1999 and subsequently incorporated into the WHO classification of thoracic tumors [1,2]. Since its initial description, fewer than 100 cases have been reported in the literature, with a female predominance and a peak incidence in the fifth to seventh decades of life [3,4].

Clinically, MNT is almost invariably asymptomatic, presenting as an incidental finding on chest imaging in the absence of pain. Unlike other thymoma subtypes, particularly B2 and B3, MNT is not associated with myasthenia gravis or other paraneoplastic syndromes [4,5]. The patient's psychiatric comorbidity and recent DVT, while clinically relevant for perioperative management, are incidental and not features previously reported in association with this tumor type.

Histologically, the hallmark of MNT is its biphasic architecture: discrete micronodules of bland epithelial cells embedded within a richly lymphoid stroma containing reactive follicles with germinal centers [2,6]. This prominent lymphoid component, comprising CD20-positive B cells in follicles and CD3-positive, TdT-negative mature T cells in the interfollicular stroma, is reactive in nature and accounts for the striking resemblance to extranodal lymphoid proliferations. The presence of TdT-positive immature T lymphocytes within the epithelial nodules is a key immunohistochemical feature confirming the thymic origin of the lesion [6,7].

Immunohistochemistry is indispensable for accurate diagnosis. The epithelial cells are consistently positive for pan-cytokeratin and p63, confirming their thymic epithelial lineage. Negativity for CD5 is useful in excluding thymic carcinoma, which is typically CD5-positive [8]. The differential diagnosis of MNT encompasses several entities. Type A thymoma shares spindle cell morphology but lacks the prominent lymphoid follicular stroma and micronodular architecture. Type AB thymoma may exhibit focal areas resembling MNT but shows a more organotypic lobular architecture without true follicular hyperplasia. Extranodal marginal zone lymphoma (mucosa-associated lymphoid tissue (MALT) type) can closely mimic MNT, given the exuberant follicular lymphoid component; however, MNT lacks clonal B-cell proliferation, and the epithelial nodules are cytokeratin-positive. Metaplastic thymoma features alternating epithelial and spindle cell areas without a follicular lymphoid stroma. Thymic carcinoma is distinguished by significant cytological atypia, high mitotic activity, and CD5 positivity [4,8].

Micronodular thymoma with lymphoid stroma generally carries an excellent prognosis. Most reported cases present at Masaoka-Koga stage I or II at diagnosis, reflecting the encapsulated and non-invasive nature of this tumor [9]. Complete surgical resection (R0) is the cornerstone of treatment and is curative in the vast majority of patients. Recurrence and distant metastasis are exceptionally rare [3]. In our patient, R0 resection was achieved with negative surgical margins and no capsular invasion. No adjuvant therapy was deemed necessary. Long-term follow-up with periodic imaging remains advisable given the limited data on recurrence in the literature.

## Conclusions

Micronodular thymoma with lymphoid stroma is a rare but diagnostically important thymic neoplasm. Its distinctive morphological features, epithelial micronodules embedded within a reactive lymphoid stroma with germinal centers, combined with a characteristic immunohistochemical profile, are diagnostic and allow differentiation from lymphoma and other thymoma subtypes. Complete surgical resection is curative, and the prognosis is excellent. Awareness of this entity among pathologists is essential to ensure accurate diagnosis and appropriate clinical management.
